# Recent Progress of Helicene Type Hole-Transporting Materials for Perovskite Solar Cells

**DOI:** 10.3390/molecules28020510

**Published:** 2023-01-04

**Authors:** Jijitha Vailassery, Shih-Sheng Sun

**Affiliations:** 1 Institute of Chemistry, Academia Sinica, Taipei 115, Taiwan; 2 Taiwan International Graduate Program, Sustainable Chemical Science and Technology, Academia Sinica, Taipei 115, Taiwan; 3 Department of Applied Chemistry, National Yang Ming Chiao Tung University, Hsinchu 300, Taiwan

**Keywords:** helicenes, hole transporting materials, perovskite solar cells, hole mobility

## Abstract

Perovskite solar cells have emerged as one of the most promising photovoltaic technologies for future clean energy sources to replace fossil fuels. Among the various components in a perovskite solar cell, the hole-transporting materials play significant roles in boosting device performance and stability. Recently, hole-transporting materials with helicene cores have received much attention due to their unique properties and ability to improve the performance and stability of the perovskite solar cells. The focus of this review is on the emerging special class of HTMs based on helicenes for perovskite solar cells. The optical, electrochemical, thermal and photovoltaic properties of helicene-based small molecules as HTMs or interfacial layer materials in n-i-p or p-i-n type perovskite solar cells are summarized. Finally, perspectives for the future development of helicene type hole-transporting materials are provided.

## 1. Introduction

Energy demand is increasing day by day to meet the needs of, and provide a comfortable life for, the growing world population. Many researchers are working towards the development of clean, green and renewable energy resources as alternatives to the non-renewable fossil fuels. Sunlight is abundant in nature and one of the best choices of renewable energy sources to relay for the development of energy conversion technology. Photovoltaic cells, by converting photon energy to electrical energy, could be the best alternative to the fossil fuels since they are effective in reducing greenhouse gas emissions. Among the various types of photovoltaic technologies, the perovskite solar cell (PSC), considered as an alternative for the conventional silicon-based solar cells, is one of the most promising candidates. The metal halide perovskite was first introduced as the sensitizer in a solar cell by Miyasaka et al., in 2009 [1]. The power conversion efficiency was 3.8% for the device due to the instability and the rapid destruction of perovskite by the liquid electrolyte. In 2012, Parks and Grätzel developed a device with spiro-OMeTAD penetrated mesoscopic TiO_2_ film and perovskite having an efficiency of 9.7% [2]. The invention of solid-state organic hole conductor to the system by Snaith et al. and Grätzel et al. led to the improvement of device performance to a range of 10% [3]. Later, the device efficiencies were enhanced over 25% by different research outputs [4]. There was a tremendous improvement in the photoconversion efficiency from 3.8 to 25.7% in a decade or so, which is unprecedented for other third generation photovoltaics. The light-absorbing material in lead halide PSCs is perovskite. The unique properties of perovskite, including a high molar extinction coefficient (1.5 × 10^4^ cm^−1^M^−1^ at 550 nm) [1], wide absorption spectral coverage, good charge carrier mobility (66 cm^2^ V^−1^ s^−1^), low exciton binding energy (<10 meV) [5], and long diffusion length for charge carriers (up to 1 µm) render the PSC a highly promising next-generation photovoltaic technology [6]. Active research is being conducted to ameliorate the performance and stability of PSCs, either by developing new charge transporting materials, introducing different components to the conventional perovskite, or by alternate and improved fabrication techniques.

**Figure 1:**
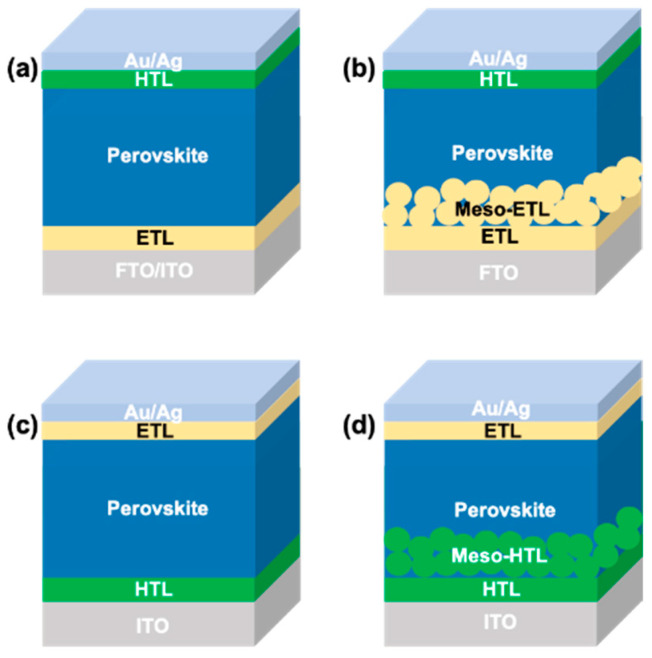
(**a**) Device structure of planar n-i-p PSCs; (**b**) Device structure of mesoporous n-i-p PSCs; (**c**) Device structure of planar p-i-n PSCs; (**d**) Device structure of mesoporous p-i-n PSCs.

**Figure 2:**
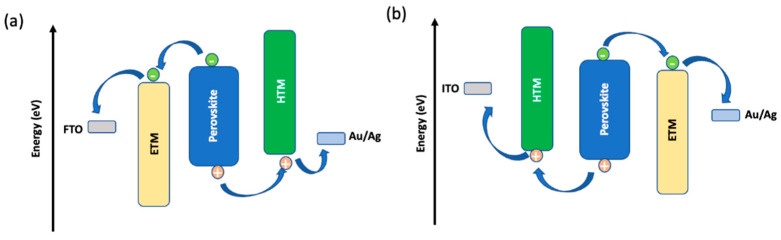
(**a**) Charge transfer process in n-i-p device; (**b**) Charge transfer process in p-i-n device.

The general working principle of a PSC is as follows: (a) formation of electron-hole pair upon photo irradiation, (b) electron transfer to the electron transporting layer (ETL), (c) hole extraction from perovskite to the hole transporting layer (HTL), and (d) hole injection into the contact electrode. There are possible undesirable charge transfer processes due to the recombination of photogenerated electrons and holes at interfaces of ETL/perovskite/HTL. Two main types of devices, namely n-i-p (conventional) and p-i-n (inverted), are possible for the PSCs due to the ambipolar charge transfer property of perovskite [7,8]. The devices are further classified into mesoporous and planar structures, thus, generally there are four types of device configuration available for PSCs, as shown in Figure 1. The first type is the mesoporous n-i-p configuration, in which a mesoporous ETL is sandwiched between the compact ETL and perovskite layer. The device fabrication method for the mesoporous n-i-p device is generally energy consuming and not suitable for the flexible substrates since the temperature condition for the processing is as high as 450 °C, which cannot withstand by a flexible substrate [9]. The second type is the planar n-i-p configuration without a mesoporous layer, often necessary for a high conductive layer such as tin oxides to harvest the electrons generated by photoirradiation deep inside the perovskite [10,11]. The third and fourth types are the inverted mesoporous p-i-n and planar p-i-n configurations where the position of ETL and HTL are interchanged in corresponding n-i-p devices. Schematic illustrations of charge transfer processes in PSCs are demonstrated in Figure 2.

Hole-transporting materials (HTMs) play significant roles in photoconversion efficiency as well as in the device stability in the conventional n-i-p PSCs [12]. A few criteria should be fulfilled by materials acting as the HTMs in a PSC. The properties of ideal HTMs should include (1) a matching HOMO level with the valence band edge of perovskite, (2) excellent hole mobility and conductivity, (3) minimum absorption in the visible and near IR region of solar spectrum, (4) photochemical and thermal stability, (5) facile synthesis and purification, and (6) good solubility in common organic solvents for the device fabrication [13]. There are three main classes of solid-state HTMs such as inorganics, polymers, and small organic molecules. The CuI, NiO_x_, and CuSCN, etc. belong to the class of inorganic HTMs. Even though these are low-cost materials with generally good hole mobility, the partial solubility of perovskite in the solvent used for the deposition of the HTMs can adversely affect the stability of the device [14]. Poly[bis(4-phenyl)(2,4,6-trimethylphenyl)amine] (PTAA) is one of the commonly used polymeric HTMs in p-i-n PSCs [15]. The polymer materials, however, suffer from the tedious purification process, low solubility, and tricky characterization with broad molar mass distribution, and issues of batch-to-batch reproducibility. The advantages of choosing small molecules as the HTMs are the ease of synthesis and characterization as well as their tunable properties. In the group of small-molecule based HTMs, spiro-OMeTAD is the most popular one and being commonly employed as the benchmark HTM for PSCs [16]. There are several drawbacks, however, for spiro-OMeTAD, including tedious synthetic and purification steps, high cost, and intrinsic low hole mobility in the order of 10^−5^ cm^2^V^−1^s^−1^. Thus, it is desirable to develop alternative HTMs with low cost of production and appropriate properties capable of significantly improving the efficiency and stability of devices. Different molecular frameworks with spiro-type core [17,18,19,20,21], D-π-D [22,23,24,25], D-A-D [26,27], and helicenes [28,29,30,31,32,33,34,35,36,37,38,39,40,41] have been explored to evaluate their applicability as HTMs for n-i-p PSCs.

**Figure 3:**
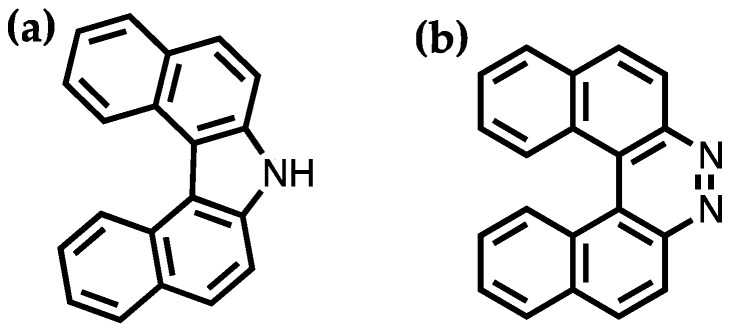
(**a**) 7*H*-dibenzo[*c*,*g*]carbazole. (**b**) benzo[*f*]naphtho [2,1-*c*]cinnoline.

Helicenes are polycyclic aromatic compounds with ortho-fused aromatic rings to have a nonplanar screw shaped skeletal arrangement [42]. Meisenheimer and Witte synthesized the first two helicenes, 7*H*-dibenzo[*c*,*g*]carbazole and benzo[*f*]naphtho [2,1-*c*]cinnoline, in 1903 as shown in Figure 3 [43]. In comparison to their planar polycyclic aromatic counterparts, the peculiar properties of helicenes mainly arise from their unique helical geometry due to the steric hindrance of the terminal rings. This allows helicenes to exhibit chiral forms without asymmetric carbons or chiral centers. According to the helicity rule, the left-handed helix is denoted by *M* and the right-handed helix is denoted by *P*. Helicenes spiral up to form cylindrical structures with a constant pitch as the number of fused rings increases. Helicenes are good π electron donors similar to other polycyclic aromatic compounds but show greater departure from planarity. In crystal forms, the interactions other than π-π interaction such as CH-π, S-S, hydrogen bonding interactions, etc., were identified [42]. The dihedral angle or interplanar angle between the two terminal rings varies depending on the different substituents on the structure or the length of helicene backbone. The dihedral angle between the terminal rings in a [11] helicene is very small, of 4°, whereas it is as large as 58.5° in a [6] helicene. A trend of reduced interplanar angles is observed as the terminal rings are substituted by short alkyl chains. Generally, the solubility of helicenes is better than their planar polycyclic aromatic counterparts. The solubility of helicenes could be further improved by introducing proper substituents such as alkyl or alkoxy groups.

**Figure 4:**
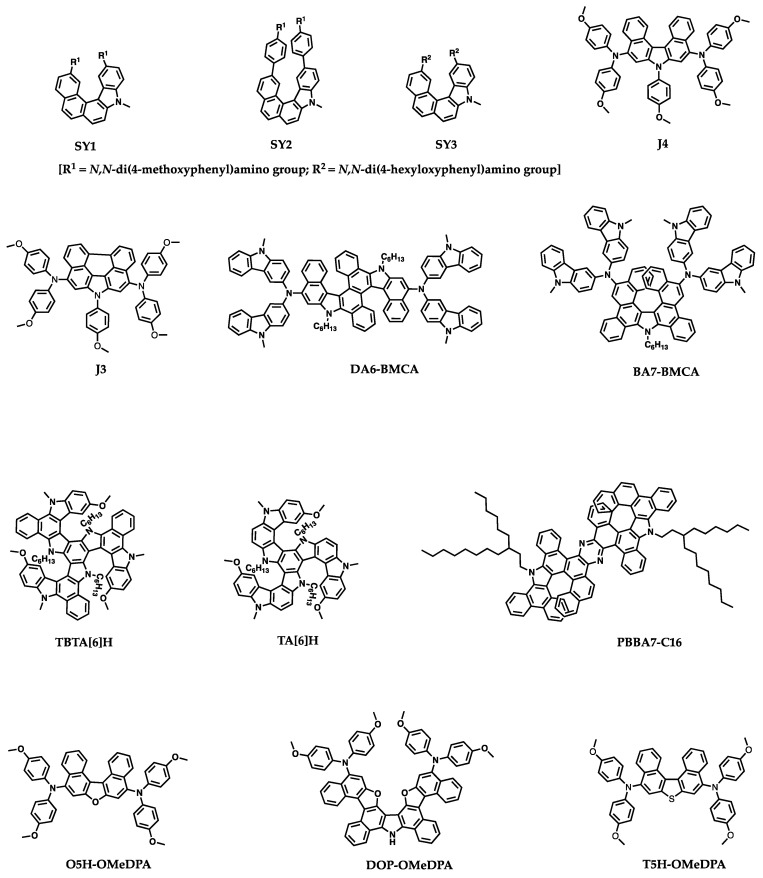

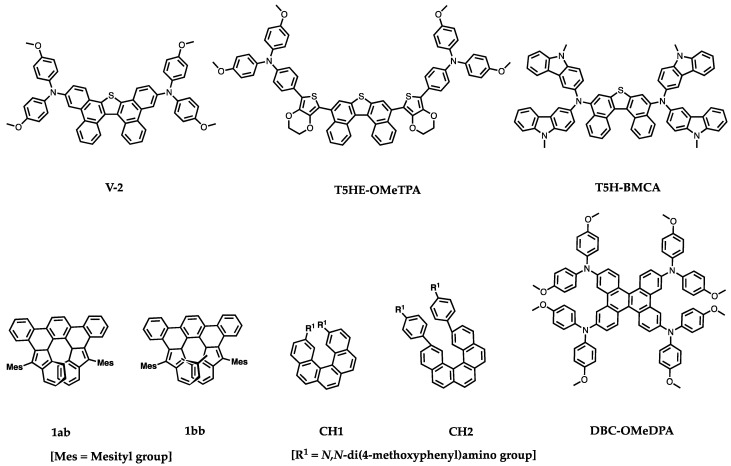
Molecular structure of HTMs.

**Figure 5:**
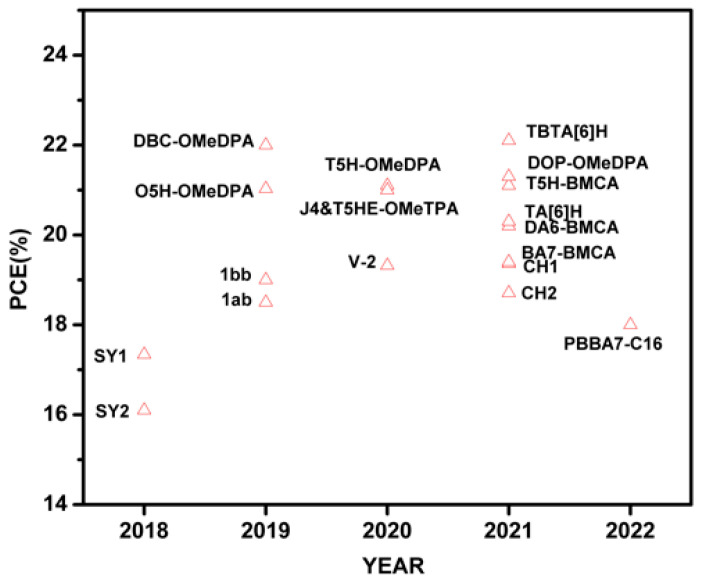
PCEs of the PSCs fabricated with helicene-based HTMs.

Recently, HTMs with helicene cores for PSCs have received wide attention because of their unique properties and ability to enhance the performance and stability of the devices. Despite several reviews detailing the synthesis, general properties, and applications of helicenes, there is no review particularly focused on the topic of helicenes utilized in PSCs. In this regard, we have attempted to summarize the optical, electrochemical, thermal and photovoltaic properties of helicene-based small-molecule HTMs or interfacial layer materials used in n-i-p or p-i-n PSCs. We have categorized the reported helicene materials based on the presence of heteroatom such as azahelicene, oxahelicene, thiahelicene, carbohelicene, double helicene to make the discussion flow convenient. The molecular structures of HTMs covered in this review are collected in Figure 4. Table 1 and Figure 5 summarize the relevant PSC performance data fabricated with helicene-based HTMs. The related details are reviewed in the following sections.

## 2. Azahelicenes in PSCs

**Figure 6:**
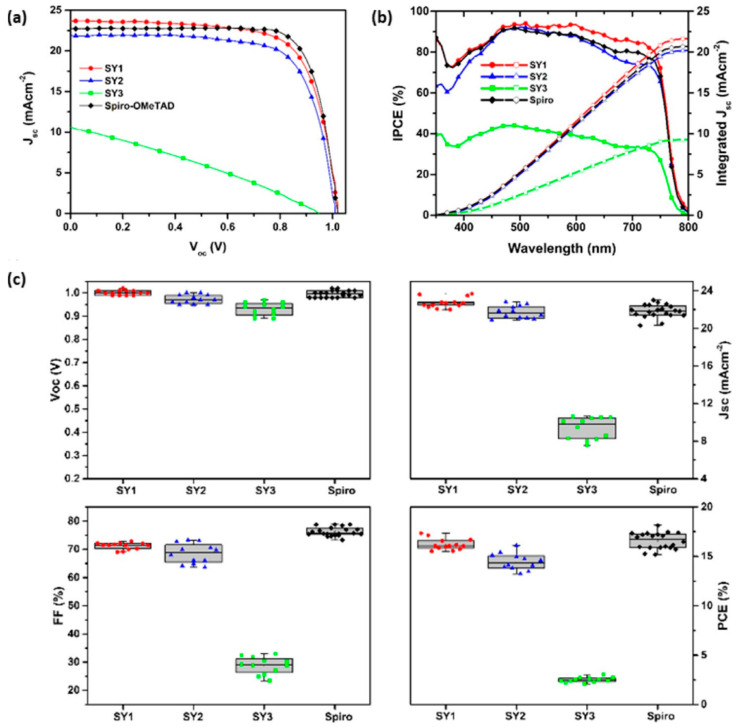
(**a**) J−V curves of PSCs with SY1, SY2, SY3, and spiro-OMeTAD recorded from reverse bias to short-circuit conditions under AM 1.5 G irradiation (100 mW cm^−2^); (**b**) IPCE plots and integrated current densities for the PSCs in (**a**); (**c**) box chart comparison of photovoltaic parameters for PSCs. Reprinted (adapted) with permission from Ref. [28]. Copyright (2018) American Chemical Society.

The first helicene-based HTMs for perovskite solar cells were reported in 2018 [28]. Three azahelicenes, namely SY1, SY2, and SY3, consisting of monoazahelicene core and electron donating terminal groups such as bis(4-methoxyphenyl)-amino or bis(*p*-methoxyphenyl)aminophenyl groups were reported for PSCs (see Figure 4 for the structures). The optical properties, thermal stability, energy level alignments with the valence band edge of perovskite, film morphology, hole mobilities and hole extraction abilities of these HTMs were investigated. The molecule SY1 showed a good thermal stability with a decomposition temperature T_d_ = 388 °C. Good morphological stability was proved by the differential scanning calorimetry (DSC) studies. The HOMO level (−4.82 eV) of SY1 was the highest among all three molecules in this study as well as higher than the HOMO of standard spiro-OMeTAD. Interestingly, the hole mobilities of SY1 and SY2 measured from the hole-only devices were higher than that of spiro-OMeTAD. However, SY3 showed very low hole mobility in the order of 10^−7^ cm^2^V^−1^s^−1^. Time-resolved photoluminescence (TRPL) decay of pristine perovskite showed a lifetime of 189.6 ns, which was reduced to 9.24 ns upon deposition of SY1 indicative of effective hole extraction from photoexcited perovskite by SY1. It was clear from the SEM images of devices that the film quality and morphology of SY1 was uniform without pinholes. The water contact angle measurements indicated the hydrophobic nature of SY1 which is helpful to prevent moisture penetration to the perovskite layer in device. A conventional perovskite CH_3_NH_3_PbI_3_ as the photoactive material was employed in this work with a configuration of FTO/c-TiO_2_/mp-TiO_2_/CH_3_NH_3_PbI_3_/HTM/Ag in PSCs. The results of relevant device performance are illustrated in Figure 6. The maximum efficiencies of 17.34, 16.10, and 3.03% were achieved by using SY1, SY2, and SY3, respectively, as the HTMs in n-i-p PSCs. SY1 showed better stability than control device in long-term stability tests and retained 97 and 80% of initial performance under storage conditions and upon the exposure to the harsh conditions. The device fabricated with SY2 as the HTM showed slightly lower efficiency than that of SY1. The devices based on SY3, which constitutes long alkoxyl chain in its structure, exhibited poor performance. The hole mobility of SY3 was much lower than the other two congeners, where the alkyl chains are likely to behave like charge barriers to hamper the intermolecular charge migration. The low T_g_ value may contribute to the instability of SY3 based device.

**Figure 7:**
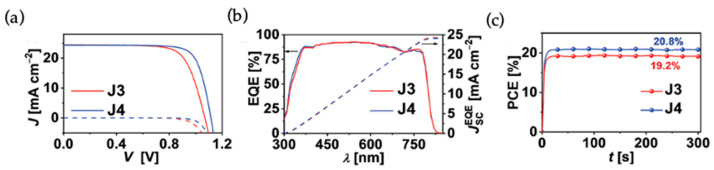
(**a**) J−V curves of PSCs with J3 (red) and J4 (blue) as the HTMs measured under the 100 mW cm^−2^, AM 1.5G illumination; (**b**) EQE spectra and integrated current density (JEQE); (**c**) PCE output via MPP tracking for PSCs. Reproduced with permission from Ref. [29]. Copyright (2020) Wiley.

Wang and coworkers reported a D-π-D type aza[5]helicene HTM for high performance PSCs [29]. In order to showcase the superior role of helicene type HTMs, they have designed two HTMs, J3 and J4, where J3 has a flat π linker perylene and J4 has a contorted π linker aza[5]helicene. Both of these molecules were end capped with the same electron donating groups, dimethoxydiphenylamine, and side functionalized with a methoxyphenyl group. The J4-based PSC with a device configuration of FTO/compact-TiO_2_/meso-TiO_2_/perovskite/HTL/Au yielded a PCE of 21% with J_sc_ = 24.31 mA cm^−2^, Voc = 1.115 V, FF = 0.774, while the devices based on J3 and spiro-OMeTAD delivered a PCE of 19.4 and 20.3%, respectively. It was noted that there are higher economic values for the J3 and J4 than spiro-OMeTAD. Most of the helicene-based compounds show excellent solubility than their planar counterparts. In this case, also the solubilities are 175 × 10^−3^ and 86 × 10^−3^ M for J4 for J3, respectively, in chlorobenzene. Good solubility of HTMs is crucial for making uniform and smooth thin films. The calculated hole mobility based on the single crystal data for J4 is 2.2 × 10^−3^ cm^2^V^−1^s^−1^, which is more than two times higher than that of J3. The average hole mobility of undoped J4 film derived from hole-only devices by space-charge limited current (SCLC) model is 4.1 × 10^−5^ cm^2^ V^−1^ s^−1^, which is four times higher than that of J3 (9.2 × 10^−6^ cm^2^ V^−1^ s^−1^). After overnight air doping in the presence of HTFSI and tBp additives, over one order higher hole mobilities for J4 and J3 were achieved as 7.1 × 10^−4^ and 2.1 × 10^−4^ cm^2^ V^−1^ s^−1^, respectively. Efficient hole extraction from the photoexcited CsMAFA by J3 and J4 HTMs were observed and the calculated yields of hole extraction values were 98.2 and 97.8%, respectively. The J3 and J4 covered perovskite film showed notably lower PL intensities than the pristine perovskite film. A stable output of 19.2% for J3 and 20.8% for J4 were observed on tracking the PCE values at the MPP for 300 s. The J-V curves, EQE spectra, and PCE output via MPP tracking are given in Figure 7. The results reported in this work demonstrated that a better performance was exhibited by the J4-based PSCs in comparison to that of its planar counterpart J3 due to its effective hole transporting properties and ability to diminish the charge recombination.

**Figure 8:**
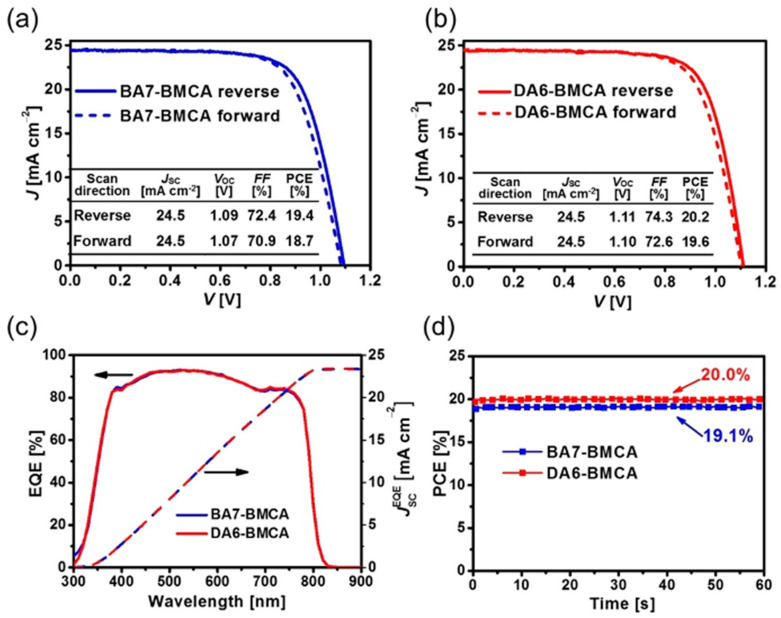
J-V characteristics of champion PSC devices based on (**a**) BA7-BMCA and (**b**) DA6-BMCA under 100 mW cm^−2^ AM1.5G sunlight; (**c**) EQE spectra and the corresponding integrated J_sc_ of PSCs employing BA7-BMCA or DA6-BMCA as HTM; (**d**) MPP tracking plots of PSCs under continuous light irradiation (AM 1.5G, 100 mW cm^−2^) at room temperature. Reproduced with permission from Ref. [30]. Copyright (2021) Wiley.

Lei and co-workers designed two helicene-type HTMs, which are polycyclic aromatic compounds with chrysene-based azahelicene as the π-linker [30]. Chrysenes are readily available and inexpensive. The structures of two helicene-based HTMs, DA6-BMCA and BA7-BMCA, are displayed in Figure 4. The performance of PSCs with the device configuration of glass/FTO/TiO_2_/perovskite/HTM/Au showed good performance of 20.2% for DA6-BMCA and a slightly lower of 19.4% for BA7-BMCA. A detailed comparison in properties of these two HTMs has been studied. DA6-BMCA showed better solubility in chlorobenzene, higher decomposition temperature, and higher glass transition temperature compared to those of BA7-BMCA. The good solubility properties of both HTMs enabled them to deposit thick layer for high performance PSCs. According to cyclic voltammetry as well as UPS investigations, the HOMO levels obtained for BA7-BMCA was slightly deeper than DA6-BMCA. The hole mobility of DA6-BMCA on the basis of SCLC model measured with a hole-only device was 1.5 times higher than that of BA7-BMCA. The hole mobility values were 4.82 × 10^−5^ and 3.03 × 10^−5^ cm^2^ V^−1^ s^−1^ for DA6-BMCA and BA7-BMCA, respectively. The hole mobilities of these HTMs were further improved to 1.45 × 10^−4^ and 1.21 × 10^−4^ cm^2^ V^−1^ s^−1^ for DA6-BMCA and BA7-BMCA, respectively, after doping of HTFSI and tBP. The HOMO energy level for BA7-BMCA is −5.09 eV, which is slightly deeper than the HOMO level of DA6-BMCA (−5.08 eV). The PLs of triple cation perovskite [(FAPbI_3_)_0.875_(MAPbBr_3_)_0.075_(CsPbI_3_)_0.05_(PbI_2_)_0.03_] deposited on meso-Al_2_O_3_ are effectively quenched after being capped with HTMs. The hole extraction yields were similar for both of the HTMs with 97.8% for BA7-BMCA and 97.5% for DA6-BMCA. A small electric hysteresis was observed in the J-V characteristics of champion devices under AM 1.5 G irradiation. The fresh cell of DA6-BMCA displayed a higher PCE of 20.2% with J_sc_ = 24.5 mA cm^−2^, Voc = 1.11 V and FF = 74.3% in the reverse scan direction. The highest PCE reported for the BA7-BMCA fresh cell was 19.4% with J_sc_ = 24.5 mA cm^−2^, Voc = 1.09 V and FF = 72.4% in the reverse scan direction (See Figure 8 for the J-V curves, EQE spectra, and MPP tracking of the BA7-BMCA and DA6-BMCA). The stability test revealed that the devices of BA7-BMCA and DA6-BMCA could maintain 81.3% and 93.1% of their initial PCEs after stored in dry air at ambient room temperature for 30 days.

**Figure 9:**
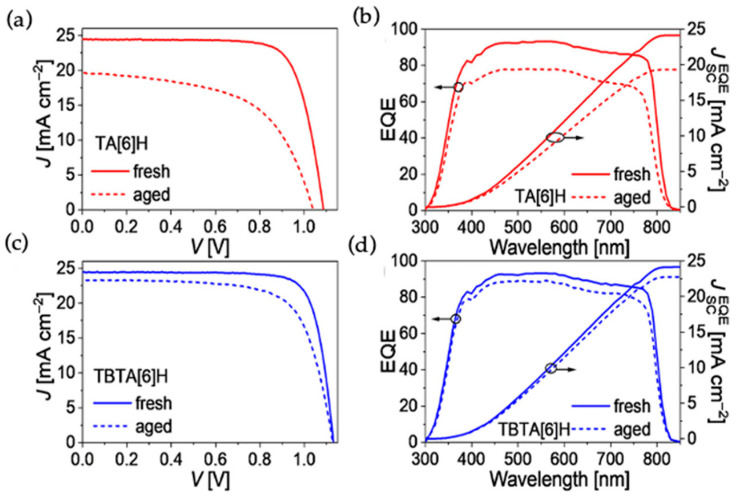
(**a**,**c**) J−V curves of PSCs based on TA[6]H and TBTA[6]H under 100 mW cm^−2^, AM1.5 G irradiation. The aged cells were stored at 85 °C for 2000 h; (**b**,**d**) EQE curves and integral J_SC_ over the standard AM1.5G spectrum. Reprinted (adapted) with permission from Ref. [31]. Copyright (2022) American Chemical Society.

In the course of pursuing high performance PSCs, it is equally important to improve the device stability. Wang and coworkers recently reported triple aza[6]helicene-based HTMs, TA[6]H and TBTA[6]H, with fully fused aromatic framework [31]. TBTA[6]H is composed of three extra benzene rings fused on TA[6]H structure and the solubilities of these materials in chlorobenzene are 264 mg mL^−1^ and 351 mg mL^−1^, respectively. The glass transition temperature (T_g_) values from DSC measurements for TBTA[6]H and TA[6]H are 195 and 168 °C, respectively, which are higher than that for spiro-OMeTAD. The same trend is followed by the estimation of T_g_ by MD simulation methods. TBTA[6]H showed an optimum T_g_ value even after doping with the BPTFSI. The HOMO levels for TBTA[6]H and TA[6]H thin films by UPS are −5.01 and −4.96 eV, respectively, which are suitable for the hole extraction from the lead halide-based alloy perovskite with valence band at −5.45 eV. The thin films of both materials showed amorphous characteristics from XRD measurements. The average hole mobility of neat TBTA[6]H (1.59 × 10^−4^ cm^2^V^−1^s^−1^) is higher than that of spiro-OMeTAD (6.99 × 10^−5^ cm^2^V^−1^s^−1^), whereas it is slightly lower for TA[6]H (5.56 × 10^−5^ cm^2^V^−1^s^−1^) by SCLC method. The improvement in hole mobilities reported up to 1 order of magnitude upon blending the dopants of BPTFSI. The hole mobilities after doping are 2.23 × 10^−3^ and 5.80 × 10^−4^ cm^2^V^−1^s^−1^ for TBTA[6]H and TA[6]H, respectively. The PCE based on dopant-free TBTA[6]H reached 20.4% (J_SC_ = 24.42 mA cm^−2^, Voc = 1.110 V, FF = 0.751) with a device configuration of glass/FTO/compact-TiO_2_/meso-TiO_2_/CsMAFA/HTL/Au. In contrast, spiro-OMeTAD based device shows an efficiency of 16.1% without dopants. The PCE of devices were enhanced on doping the HTL with BPTFSI. TBTA[6]H-based device showed an improved PCE of 22.1%, whereas the standard device with spiro-OMeTAD achieved 21.2% efficiency. The cells based on TA[6]H HTM exhibited a slightly lower PCE of 17.9% with J_sc_ = 24.41 mA cm^−2^, Voc = 1.045 V, and FF = 0.701. The PCE of the device further improved to 20.3% on doping with BPTFSI composites. TBTA[6]H-based device showed much better photostability than spiro-OMeTAD based device. After 500 h of light soaking, the TBTA[6]H-based cell retained 93% of its initial PCE at 55 °C. The TBTA[6]H-based device also showed good thermal stability. Only a loss of 15% PCE was observed for the TBTA[6]H-based cell at temperature of 85 °C and dark conditions after 2000 h. The photovoltaic parameters are illustrated in Figure 9.

**Figure 10:**
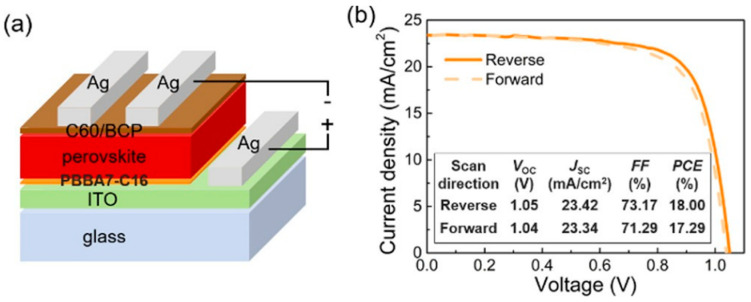
(**a**) Device architecture; (**b**) J−V curves and performance of the best device measured in reverse and forward scan modes. Reprinted (adapted) with permission from Ref. [32]. Copyright (2022) American Chemical Society.

A series of pyrazine-fused bis-aza[7]helicene (PBBA7) derivatives, synthesized by repeated tandem electro-oxidative C−C and C−N coupling and aromatization in 90.0–93.2% isolated yields, were reported by Yu et al. [32]. The PBBA7-C16 was utilized as the HTM for perovskite solar cells. The HOMO and LUMO determined by cyclic voltammetry are −5.05 and −1.97 eV, respectively. This HOMO energy level was found to be suitable for hole extraction from Cs_x_MA_y_FA_1-x-y_PbI_3_ perovskite. The PSC with an inverted p-i-n device configuration of ITO/PBBA7-C16/perovskite/C60/BCP/Ag exhibited a power conversion efficiency (PCE) of 18.00% with a short circuit current density of 23.42 mA cm^−2^, an open circuit voltage of 1.05 V, and a fill factor of 73.17% as illustrated in Figure 10 for the device structure and J-V curves of the best performing device of PBBA7-C16.

## 3. Oxahelicenes in PSCs

**Figure 11:**
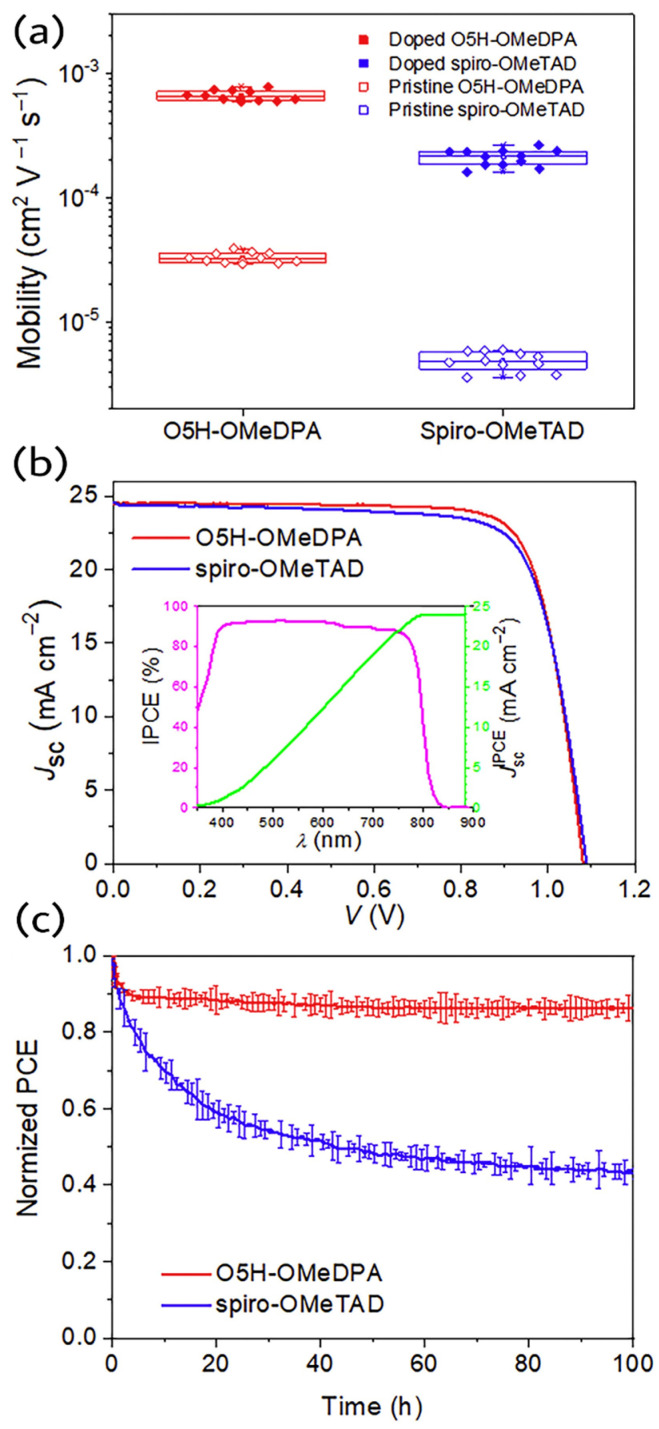
(**a**) SCLC hole mobilities of O5H-OMeDPA and spiro-OMeTAD spin coated on the substrate of poly(3,4-ethylenedioxythiophene)polystyrene sulfonate; (**b**) J-V characteristic (reverse scan) of a champion PSC with O5H-OMeDPA as the HTL measured under AM 1.5G illumination. The inset is the IPCE spectrum and integrated J_sc_ from the IPCE curve for the O5H-OMeDPA based cell; (**c**) Normalized PCEs of unencapsulated devices examined via MPP tracking under the continuous AM1.5G equivalent light irradiation and nitrogen flow at 60 °C. Error bars refer to the average deviations of four cells. Reproduced with permission from Ref. [33]. Copyright (2019) Cell Press.

Wang et al. attempted to address the durability issues under light and thermal stress of perovskite solar cells in a study by introducing a racemic semiconducting glassy film of O5H-OMeDPA with an improved morphological stability [33]. O5H-OMeDPA is an oxa[5]helicene-based low symmetrical entity with helical configuration which allows the multiple dimension of charge transfer in the solid. The hole mobility of O5H-OMeDPA estimated from single crystal data is up to 4.7 × 10^−2^ cm^2^V^−1^s^−1^ and the average hole mobility obtained from hole-only devices by SCLC model is 3.3 × 10^−5^ cm^2^V^−1^s^−1^, which is over five times higher than that of spiro-OMeTAD. Doping with TBP and LiTFSI further increases the mobility to 6.7 × 10^−4^ cm^2^V^−1^s^−1^. PSCs with a triple cation perovskite (FAPbI_3_)_0.85_(MAPbBr_3_)_0.10_(CsPbI_3_)_0.05_(PbI_2_)_0.03_ and a device configuration of FTO/c-mp TiO_2_/perovskite/HTL/Au were fabricated. A champion PSC-based on O5H-OMeDPA yielded an efficiency of 21.03%. The operation stability tests at 60 °C and 1 sun equivalent white LED illumination at MPP conditions revealed that 86% of the initial PCE was retained by the O5H-OMeDPA after 100 h. It was noticed that the PCE remained stable after the initial burn-in decay, which may be attributed to the migration of A site cations of perovskite. The hole extraction properties of control and helical HTM were almost similar. The PL quenching yields obtained after coating HTMs on top of the perovskite layer were 96.3 and 97% for spiro-OMeTAD and O5H-OMeDPA, respectively. The shorter PL lifetime of O5H-OMeDPA coated perovskite film than that of spiro-OMeTAD indicates that a stronger electronic coupling of O5H-OMeDPA with the perovskite than the spiro-OMeTAD. DFT studies also suggests the higher interface stability and thus the greater interface affinity of O5H-OMeDPA over spiro-OMeTAD. The authors critically discussed the issues related to the crystallization. If large crystalline domains in the HTL exist then the perovskite solar cells will degrade easily at elevated temperature. As revealed by XRD analysis, the microstructure of solution processed glassy film of O5H-OMeDPA remained amorphous even after the thermal stress at 60 °C whereas there were multiple diffraction patterns appeared in the case of spiro-OMeTAD after the thermal stress. The molecules in the thin film will move and form closely packed phases and crack easily during crystallization. The hole extraction and transportation may be affected by the heat-induced phase transition and resulted in deteriorated interfacial contacts. Moreover, the changes in the film morphology of hole transporting layer weakens its blocking effect on Au diffusion to perovskite layer which will badly affect the performance of device. In this context, the racemic film showed superior photo and thermal stability over spiro-OMeTAD at the conditions mentioned above. The comparison of hole mobility, device performance and stability tests of O5H-OMeDPA with spiro-OMeTAD are illustrated in Figure 11.

**Figure 12:**
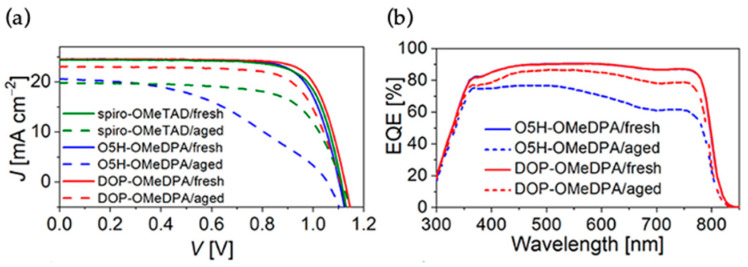
(**a**) J−V characteristics of fresh and aged PSCs measured under the 100 mW cm^−2^ AM1.5 G irradiation. (**b**) EQE curves of fresh and aged PSCs. Reprinted (adapted) with permission from Ref. [34]. Copyright (2021) American Chemical Society.

A pyrrole bridged oxa[5]helicene-based solution processable molecular semiconductor DOP-OMeDPA was reported by Wang [34]. DOP-OMeDPA is structurally fused of two oxa[5]helicenes with a pyrrole linkage. There are several advantages of DOP-OMeDPA over the single oxa[5]helicene based O5H-OMeDPA including improved hole mobility and glass transition temperature. The doped DOP-OMeDPA also shows high conductivity and slow interfacial charge recombination. The DOP-OMeDPA-based fresh cells achieved a PCE of 21.8% with J_sc_ = 24.55 mA cm^−2^, Voc = 1.130 V, and FF = 0.786. The thin film of DOP-OMeDPA showed excellent morphology stability at 60 ^0^C and retained 81% of the efficiency after 500 h aging. The photovoltaic characteristics of fresh and aged cells are given in Figure 12.

## 4. Thiahelicenes in PSCs

**Figure 13:**
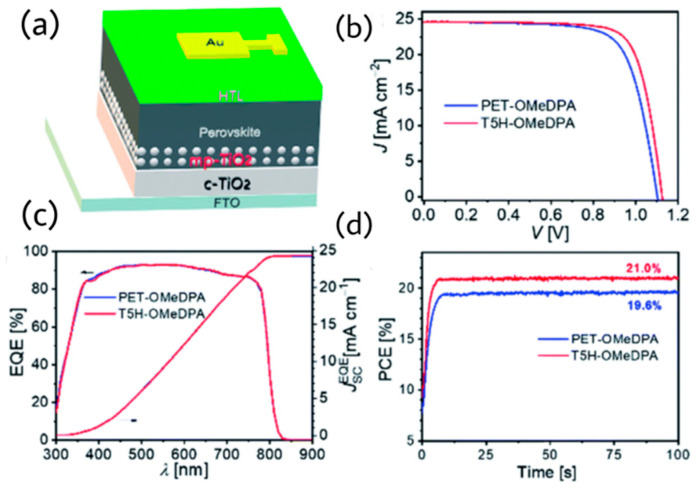
(**a**) Illustration of PSCs; (**b**) J–V curves under 100 mW cm^−2^, simulated AM1.5 G sunlight of PSCs with PET–OMeDPA and T5H–OMeDPA; (**c**) EQE curves and integrated J_sc_ over the standard AM1.5 G spectrum; (**d**) PCE output via MPP tracking. Reproduced with permission from Ref. [35]. Copyright (2020) Royal Society of Chemistry.

In contrast to the intuitive thought of facilitating intermolecular charge transport for planar organic D-π-D semiconductor, Wang et al. reported an intriguing helical organic D-π-D semiconductor, T5H-OMeDPA, with a thia[5]helicene core, which indeed exhibits better charge transporting properties than its planar perylothiophene congener, PET-OMeDPA [35]. The crystallographic analysis and computational studies of T5H-OMeDPA and PET-OMeDPA revealed that better π-π stacking was found in T5H-OMeDPA single crystal than that of PET-OMeDPA. The hole mobility of T5H-OMeDPA single crystal is about five times higher than that of PET-OMeDPA. The solution processed film of T5H-OMeDPA showed hole mobility of 6.26 × 10^−4^ cm^2^ V^−1^ s^−1^, which is three times higher than that of PET-OMeDPA. Moreover, compared to PET-OMeDPA, the weaker electronic coupling of T5H-OMeDPA with perovskite effectively suppressed interfacial charge recombination. The champion PSC fabricated based on T5H-OMeDPA exhibited a power conversion efficiency of 21.1% with J_sc_ = 24.60 mA cm^−2^, Voc = 1.125 V and FF = 0.764. In contrast, the PSC with PET-OMeDPA produced a power conversion efficiency of 19.8% with J_sc_ = 24.58 mA cm^−2^, Voc = 1.100 V and FF = 0.732. The schematic representation of device, J-V curves, EQE curves, PCE output via MPP tracking are shown in Figure 13.

**Figure 14:**
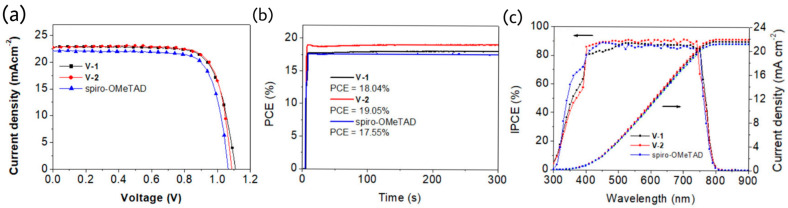
(**a**) J−V characteristics of the optimized device based on V-1, V-2, and spiro-OMeTAD as HTMs under the reverse scan. (**b**) The stabilized PCE of PSCs near the MPP voltages. (**c**) IPCE spectra and integrated current densities of PSC devices with V-1, V-2, and spiro-OMeTAD as HTMs. Reprinted (adapted) with permission from [36]. Copyright (2020) American Chemical Society.

Tao et al. reported two, hole transporting materials, V-1 and V-2, composed of diphenanthrothiophene (DPT) core unit end capped with different arylamines [36]. The crystal structure analysis and DFT calculation revealed the twisted geometry of DPT scaffold. Such fused and noncoplanar structure disturbs the cofacial packing and generates amorphous properties. V-1 has a slightly deeper HOMO level than that of V-2 by cyclic voltammetry measurements and the same trend were observed in PESA measurements and DFT calculations. Obviously, those materials were suitable to act as charge transporting materials and the higher LUMOs make them as an electron blocking layer to prevent the recombination at counter electrode. The hole mobility of V-2 was higher than those of V-1 and spiro-OMeTAD, which is attributed to the herringbone-type stacking geometry and more planar backbone for V series HTMs than spiro-OMeTAD. Better charge extraction capability of V-2 than both V-1 and spiro-OMeTAD was confirmed by the PL and TRPL studies where stronger PL quenching effect and shorter PL lifetime was identified for V-2 than those of V-1 and spiro-OMeTAD. The PCE of V-2 based devices were impressively high as 19.32% and the best efficiency for V-1 based devices was 18.60%, both were outperformed spiro-OMeTAD with an efficiency of 17.99%. The aging tests showed that V-2 based devices retained 85% PCE after 300 h at 85% relative humidity at 25 °C. The photovoltaic characteristics of both V-1 and V-2 are illustrated in Figure 14.

**Figure 15:**
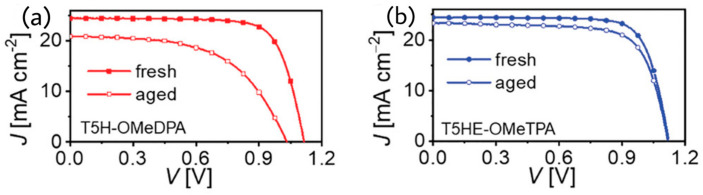
Photocurrent density−voltage curves measured for T5H-OMeDPA (**a**) and T5HE-OMeTPA (**b**) under 100 mW cm^−2^ AM1.5G irradiation. Reproduced with permission from Ref. [37]. Copyright (2021) Wiley.

Wang et al. reported a racemic molecular semiconductor, T5HE-OMeTPA, based on a thia[5]helicene core and extended π-spacer of ethylenedioxythiophene (EDOT), which exhibited 21% efficiency on application as the HTM in a perovskite solar cell [37]. The molecule of T5HE-OMeTPA is a modification of T5H-OMeDPA where an additional π-linker EDOT and phenyl ring are introduced in molecular structure. Theoretical studies were performed to reveal various electronic properties such as reorganization energies, hole transfer, energy disorders, hole transfer integrals, and centroid distances. The theoretical hole mobility µ of T5HE-OMeTPA is 1.1 cm^2^V^−1^s^−1^, whereas the theoretical µ of previously reported T5H-OMeDPA is 1.3 × 10^−2^ cm^2^V^−1^s^−1^. The hole mobility of the racemic thin film of T5HE-OMeTPA was experimentally estimated by SCLC analysis to be 2.66 × 10^−5^ cm^2^V^−1^s^−1^, which is higher than that of T5H-OMeDPA (1.28 × 10^−5^ cm^2^V^−1^s^−1^). The hole mobility of T5HE-OMeTPA was further improved on BPTFSI air doping over one order to 4.85 × 10^−5^ cm^2^V^−1^s^−1^. The PSC with an architecture of glass/FTO/compact-TiO_2_/meso-TiO_2_/perovskite/T5HE-OMeTPA/Au exhibited a PCE of 21.0% with J_sc_ = 24.43 mA cm^−2^, Voc = 1.120 V, FF = 0.767 as illustrated in Figure 15. The T5HE-OMeTPA-based cell retained 90% of initial PCE after 1000 h accelerated aging test at 60 °C in the dark.

**Figure 16:**
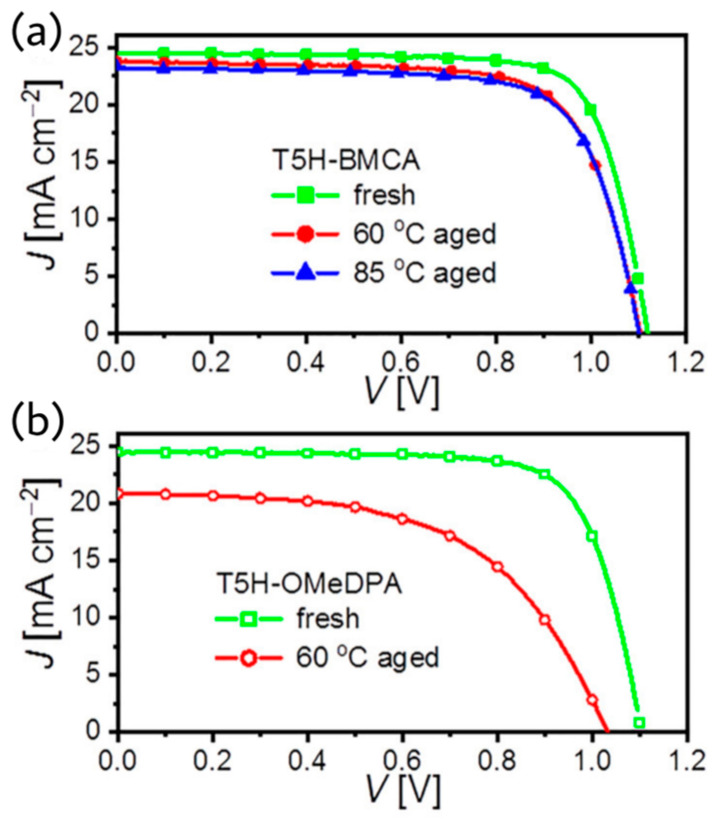
J−V curves measured for (**a**) T5H-BMCA and (**b**) T5H-OMeDPA at the 100 mW cm^−2^, AM1.5G conditions. Reprinted (adapted) with permission from Ref. [38]. Copyright (2021) American Chemical Society.

The introduction of high T_g_ organic semiconductors is always crucial for the developments of the thermostable PSCs. Inspired by the in silico screening and predictions of high T_g_ materials, Wang et al. reported a bis(9-methyl-9H-carbazol-3-yl) amine functionalized thiahelicene-based organic semiconducting material, T5H-BMCA, for perovskite solar cells [38]. T5H-BMCA enabled the corresponding perovskite device to achieve 21% efficiency, stability for 1000 h at 85 °C. The operational stability of the device under continuous full sunlight at 60 °C was found to be good. The TRPL traces showed distinct charge separation at perovskite/HTM interfaces. The hole mobilities by the SCLC measurements of pristine T5H-BMCA and air-doped thin films with dopant promoter BPTFSI are 4.66 × 10^−5^ and 5.33 × 10^−4^ cm^2^V^−1^s^−1^, respectively; both are much better than that of T5H-OMeDPA. The T_g_ values by DSC experiments were also higher for the T5H-BMCA (248 °C) than previously reported analogue T5H-OMeDPA (137 °C). However, the addition of BPTFSI resulted a decrease in Tg value of composites to 177 °C. The T5H-BMCA-based PSCs achieved a highest PCE of 21.1% with J_sc_ = 24.45 mA cm^−2^, Voc = 1.120 V, and FF = 77.1%. The T5H-BMCA-based PSCs showed 92% retention ratio of initial PCE after aging to 1000 h at 60 °C. The device characteristics at fresh condition and after aging at different temperature are shown in Figure 16.

## 5. Carbohelicenes in PSCs

**Figure 17:**
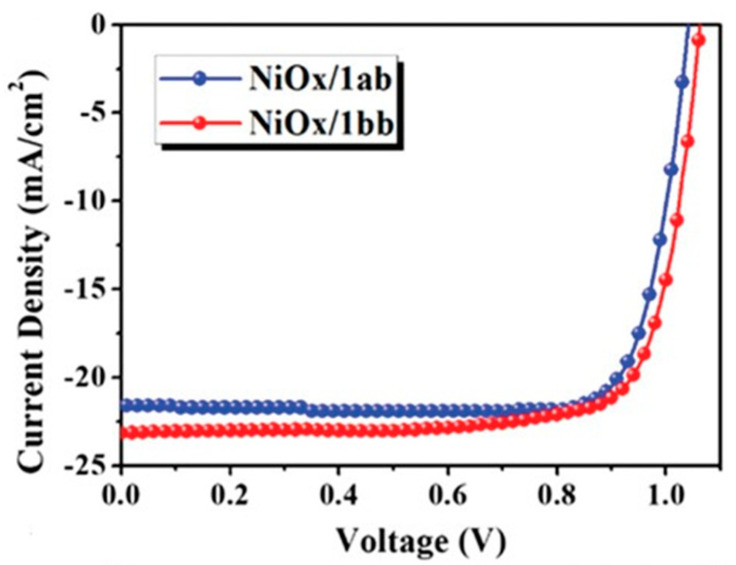
The J-V curves of the topper forming devices using NiO_x_/1ab and NiO_x_/1bb as the HTLs. Reproduced with permission from Ref. [39]. Copyright (2019) Wiley.

The inorganic NiO_x_ is one of the most commonly used HTMs in p-i-n devices. However, the surface defects on NiO_x_ lead to a negative effect on the quality of the perovskite film grown on top of NiO_x_ and the subsequent hole extraction and charge transfer process. Chueh et al. reported two [7]helicenes, 1ab and 1bb, with stable biradical character as the hole extraction layers for the p-i-n perovskite solar cells to improve the performance [39]. These materials are special since they possess open-shell singlet biradical ground states at room temperature with excellent chemical and thermal stability. The structure of 1bb is more twisted than that of 1ab since the former possesses a bulkier methyl group. The studies of film quality revealed that these helicene molecules can effectively smoothen the rough surface of NiO_x_ by passivating the associate boundaries. The surface morphology of perovskite was very clear from the SEM measurements that the large crystal size of perovskite grown on 1ab and 1bb than pristine NiO_x_ was evident. Both 1ab and 1bb showed better hydrophobic character than the NiO_x_. The water contact angles of the helicenes of this study are greater than 81° whereas NiO_x_ is only 38.94 °. The average grain size for perovskite film were 268.6, 260.6, and 192.1 nm, for 1ab modified film, 1bb modified film, and pristine NiO_x_ film, respectively. The improved crystallinity of perovskite films leads to suppression of charge recombination arising from the defects and grain boundaries. The J-V curves of the best performing devices are illustrated in Figure 17. The study demonstrates that this surface modified devices by helicenes outperformed the control device with sole NiO_x_ HTL. NiO_x_/1ab based devices achieved an efficiency of 18.5% with a J_sc_ = 21.58 mAcm^−2^, V_oc_ = 1.028 V and FF = 82.2%, whereas the NiO_x_/1bb-based devices achieved a slightly higher PCE_max_ of 19% with J_sc_ = 23.17 mA cm^−2^, Voc = 1.055 V and FF = 77.4%.

**Figure 18:**
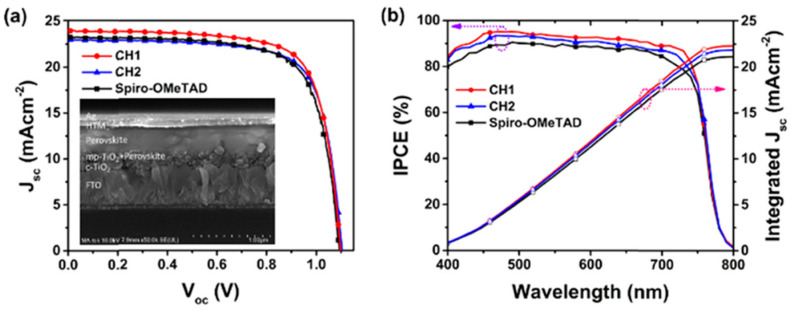
(**a**) J−V characteristics of the PSCs employing CH1, CH2, and Spiro-OMeTAD as HTMs measured under reverse scan and (**b**) their corresponding (IPCE) spectra. Reprinted (adapted) with permission from Ref. [40]. Copyright (2021) American Chemical Society.

In addition to the helicenes with heteroatoms, the carbohelicene without any heteroatoms also gained attention in this research field. The carbohelicene derivatives CH1 and CH2 were reported as the hole transporting material for n-i-p perovskite solar cell by Sun et al. [40]. In this study, they have illustrated the CH-π interactions present in crystal packing of these materials which differentiate them from the π-π interactions typically presented in planar molecules. Both CH1- and CH2-based devices exhibited superior performance than the control device based on spiro-OMeTAD. Perovskite devices in which a triple cation perovskite with formula Cs_0.05_FA_0.79_MA_0.16_PbI_2.49_Br_0.51_ as the photoactive layer and CH1 as the hole transporting layer achieved a maximum efficiency of 19.36% with J_sc_ = 23.98 mA cm^−2^, Voc = 1.098 V and FF = 73.53%. The maximum performance showed by the devices based on CH2 is 18.71% with a J_sc_ = 22.96 mA cm^−2^, Voc = 1.104 V and FF = 73.79%. The stability tests further confirmed the device durability. The photovoltaic characteristics of CH1 and CH2 in comparison with spiro-OMeTAD are illustrated in Figure 18. The PCE of devices based on the CH1 and CH2 were maintained at 90% of the initial performance after 500 h in a storage condition of 50–60% relative humidity at 25 °C.

## 6. Double Helicene in PSCs

**Figure 19:**
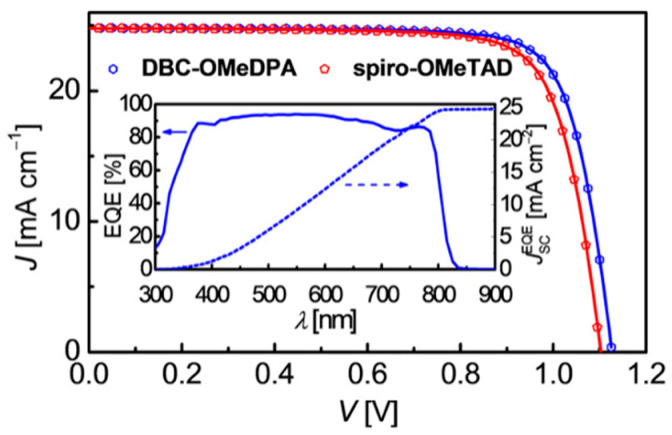
J−V characteristics of typical PSCs with DBC-OMeDPA and spiro-OMeTAD as HTMs measured under 100 mW cm^−2^, AM 1.5G illumination. The dotted data are experimentally measured, and the solid lines are fittings by the Shockley equivalent circuit. The inset is the EQE spectrum and integrated J_sc_ from the EQE curve for the DBC-OMeDPA-based cell. Reprinted (adapted) with permission from Ref. [41]. Copyright (2019) American Chemical Society.

Wang and coworkers reported a double [4]helicene-based molecular semiconductor DBC-OMeDPA [41]. The perovskite solar cells with DBC-OMeDPA as the HTM showed a PCE of 22% and a stable PCE output for hundreds of hours at 60 °C under simulated sunlight soaking. DBC-OMeDPA was appeared as in double blade propeller shape by X-ray crystallographic analysis. This molecule was crystallized in monoclinic space group P2(1)/c with eight molecules of DBC-OMeDPA in a unit cell. They conducted theoretical calculations such as QTAIM, EDA on six dimers in single crystal of DBC-OMeDPA to interpret the non-covalent interactions NCIs. DBC-OMeDPA showed four times higher hole mobility than spiro-OMeTAD and the average hole mobility of DBC-OMeDPA film is 8.8 × 10^−4^ cm^2^ V^−1^ S^−1^. This double helicene-based device exhibited superior efficiency than the control device by spiro-OMeTAD in an n-i-p PSC with a configuration of glass/FTO/TiO_2_/perovskite/HTM/Au. Both J-V curves and EQE spectrum of devices based on DBC-OMeDPA and spiro-OMeTAD are given in Figure 19. The highest PCE achieved by the DBC-OMeDPA is 22% with J_sc_ = 24.8 mA cm^−2^, Voc = 1.13, and FF = 0.79. Another merit of this HTM is the high T_g_ value, which significantly contributed to the device stability under double stress conditions of heat and electrical field. The T_g_ values for pristine DBC-OMeDPA and doped DBC-OMeDPA are 154 and 102 °C, respectively, and the PSCs retained 85% of its initial PCE even after 500 h at 60 °C.

## 7. Conclusions and Future Perspectives

This review provides a brief summary of recent progress on emerging class of helicene type molecules in both n-i-p and p-i-n devices as either hole transporting materials or interfacial layer materials. The molecular structure design of helicene-type molecules and their physical characteristics as HTMs in PSCs have been presented. Despite the number of works reported in literature with helicene type molecules are much less in comparison to the other structural types of HTMs, the discussions point out that the helicene type materials in PSCs could be very promising while considering many aspects. Most of the helical HTMs mentioned above exhibit high hole mobility as it required. Interestingly, the three-dimensional structure of helicenes facilitate the charge transport in multiple dimensions, especially with donor–acceptor structural design, which leads to stronger intermolecular interactions to further enhance hole mobility. The variation of donor–acceptor structural design is also allowed to easily modify the HOMO levels of HTMs to match with the valence band edge of perovskite. This makes an effective hole extraction and transportation process. In addition, high quality films of hole transporting layer could be formed from the helicene type materials as they show good solubility in solvents such as chlorobenzene, which is advantageous for solution processibility. Generally, the majority of these twisted HTMs with structural rigidity provide high thermal stability as evident from the T_g_ and T_d_. The morphological stability of hole transporting layer is an advantage and it resists the degradation of photoactive layer to certain extend. Some of the reports above already demonstrated that the high efficiency PSCs with helicene-type materials can withstand various stress conditions of heat and light without diminishing the PCE drastically. Most of the examples of helical materials discussed above are excellent HTM candidates in comparison with its planar counterpart or standard spiro-OMeTAD.

In case of device performance, TBTA[6]H-based device exhibited highest efficiency (22.1%) in the group of azahelicenes in PSCs which has highest hole mobility of 2.23 × 10^−3^ cm^2^V^−1^s^−1^. DOP-OMeDPA from the class of oxahelicenes in PSCs and T5H-BMCA from the thiahelicenes in PSCs achieved highest PCE of 21.8 and 21.1%, respectively. Molecule 1bb used as the interfacial layer to modify the HTM layer and exhibited a PCE of 19% in a p-i-n device. Carbohelicene CH1 showed a best efficiency of 19.36% in an n-i-p PSC. DBC-OMeDPA which belongs to the category of double helicene, is also one of the promising HTM with 22% PCE.

Recent reports highlighted that helicene-type materials are capable of enhancing the efficiency and stability of perovskite solar cells. However, some of the versatile helicene-type materials require tedious synthetic steps with low total yields. This will limit their large-scale application and commercialization. Anyway, many of them could be synthesized from readily available starting materials with reduced cost which is always beneficial for the commercialization of PSCs. It is still highly desirable to develop dopant-free HTMs by improving the hole mobility and molecular conductivity. It is much appreciated that cost effective as well as green synthetic protocols are developed to prepare these functional materials. Since it is important to optimize each component in device structure to enhance the performance and durability of PSCs, we believe that there is still enough room for the research focusing on the helicene-type materials in PSCs.

## Figures and Tables

**Table 1:** The photoelectric properties and device structure of PSCs based on helicene-based HTMs.

HTM	HOMO/ LUMO [eV]	Hole Mobility [cm^2^ V^−1^ s^−1^]	Device Structure	*Voc* [V]	*J*_sc_ [mA cm^−2^]	FF [%]	PCE [%]	Ref.
SY1	−4.82/−2.08	2.55 × 10^−5^	FTO/c-TiO_2_/mp-TiO_2_/CH_3_NH_3_PbI_3_/HTM/Ag	1.01	23.68	72.12	17.34	[28]
SY2	−4.95/−1.94	3.46 × 10^−5^	FTO/c-TiO_2_/mp-TiO_2_/CH_3_NH_3_PbI_3_/HTM/Ag	1	21.87	73.23	16.10	[28]
SY3	−4.94/−1.95	2.93 × 10^−7^	FTO/c-TiO_2_/mp-TiO_2_/CH_3_NH_3_PbI_3_/HTM/Ag	0.94	10.56	30.48	3.03	[28]
J4	−5.21/	7.1 × 10^−4^	FTO/c-TiO_2_/mp-TiO_2_/CsMAFA/HTM/Au	1.115	24.31	77.4	21.0	[29]
BA7-BMCA	−5.09/−2.46	1.21 × 10^−4^	Glass/FTO/TiO_2_/(FAPbI_3_)_0.875_(MAPbBr_3_)_0.075_(CsPbI_3_)_0.05_(PbI_2_)_0.03_/HTM/Au	1.09	24.5	72.4	19.4	[30]
DA6-BMCA	−5.35/−2.76	1.45 × 10^−4^	Glass/FTO/TiO_2_/(FAPbI_3_)_0.875_(MAPbBr_3_)_0.075_(CsPbI_3_)_0.05_(PbI_2_)_0.03_/HTM/Au	1.11	24.5	74.3	20.2	[30]
TBTA[6]H	−5.01/	2.23 × 10^−3^	FTO/c-TiO_2_/mp-TiO_2_/CsMAFA/HTM/Au	1.130 ± 0.005	24.43 ± 0.04	80.0 ± 0.4	22.1± 0.2	[31]
TA[6]H	−4.96/	5.80 × 10^−4^	FTO/c-TiO_2_/mp-TiO_2_/CsMAFA/HTM/Au	1.090 ± 0.006	24.44 ± 0.04	76.2 ± 0.5	20.3 ± 0.2	[31]
PBBA7-C16	−5.05/−1.97	-	ITO/HTM/Perovskite/ETL/Ag	1.05	23.42	73.17	18	[32]
O5H-OMeDPA	−5.29/	6.7 × 10^−4^	FTO/c-mp-TiO_2_/(FAPbI_3_)_0.85_(MAPbI_3_)_0.05_ (PbI_2_)_0.03_/HTM/Au	1.081	24.48	78.8	21.03	[33]
DOP-OMeDPA	−5.19/−2.53	6.5 × 10^−5^	FTO/TiO_2_/Perovskite/HTM/Au	1.130	24.55	78.6	21.8	[34]
T5H-OMeDPA	−5.28/−2.69	6.26 × 10^−4^	FTO/c-TiO_2_/mp-TiO_2_:CsMAFA/CsMAFA/HTM/Au	1.125	24.60	76.4	21.1	[35]
V-2	−5.24/−2.54	4.32 × 10^−4^	FTO/c-TiO_2_/mp-TiO_2_/CH_3_NH_3_PbI_3_/HTM/Ag	1.09	22.72	78	19.32	[36]
T5HE-OMeTPA	−5.22/−2.86	4.84 × 10^−4^	FTO/c-TiO_2_/mp-TiO_2_/perovskite/HTM/Au	1.120	24.43	76.7	21.0	[37]
T5H-BMCA	−4.91/−2.53	5.33 × 10^−4^	FTO/c-TiO_2_/mp-TiO_2_/Perovskite/HTM/Au	1.120	24.45	77.1	21.1	[38]
NiO_x_/1ab	−5.3/−3.9	-	ITO/NiO_x_/1ab/Perovskite/PCBM/BCP/Ag	1.041	21.58	82.2	18.5	[39]
NiO_x_/1bb	−5.0/−3.5	-	ITO/NiO_x_/1bb/Perovskite/PCBM/BCP/Ag	1.055	23.17	77.4	19.0	[39]
CH1	−5.25/−2.33	1.33 × 10^−5^	FTO/c-TiO_2_/mp-TiO_2_/CsFAMA/HTM/Ag	1.098	23.98	73.53	19.36	[40]
CH2	−5.37/−2.50	1.05 × 10^−5^	FTO/c-TiO_2_/mp-TiO_2_/CsFAMA/HTM/Ag	1.104	22.96	73.79	18.71	[40]
DBC-OMeDPA	−5.26/	8.8 × 10^−4^	FTO/TiO_2_/Perovskite/HTM/Au	1.13	24.8	79	22	[41]

## Data Availability

Not applicable.
